# Advances in Microwave-Assisted Production of Reduced Graphene Oxide

**DOI:** 10.3389/fchem.2019.00355

**Published:** 2019-06-04

**Authors:** Xinxin Xie, Yanping Zhou, Kama Huang

**Affiliations:** ^1^College of Electronics and Information Engineering, Sichuan University, Chengdu, China; ^2^Key Laboratory of Wireless Power Transmission of Ministry of Education, Sichuan University, Chengdu, China

**Keywords:** microwave-assisted synthesis, reduced graphene oxide, chemical reduction, thermal reduction, exfoliation of graphite oxide

## Abstract

Efficient reduction of graphene oxide to obtain high-quality graphene nanosheets is desirable for energy storage, catalysis, electronics and environmental remediation. In this brief review, we mainly focus on the microwave-assisted production of reduced graphene oxide in three categories: (1) microwave-assisted chemical reduction of graphene oxide; (2) microwave-assisted thermal reduction of graphene oxide; (3) microwave-assisted simultaneous thermal exfoliation & thermal reduction of graphite oxide. We also summarize common techniques for characterizing reduction efficiency and quality of as-obtained rGO.

## Introduction

Graphene is a two-dimensional sheet of sp2-hybridized carbon. Owing to its theoretically high mechanical strength (1,060 GPa), thermal conductivity (~5,000 W m^−1^ K^−1^), electron mobility (2 × 105 cm^2^ V^−1^ s^−1^), Young's modulus (~1 TPa), surface area (2,630 m^2^ g^−1^), electrical conductivity (~2,000 S m^−1^), and extraordinary optical properties, graphene has been attracting increasing application in various areas including energy storage [like fuel cells (Li et al., 2012; Hur and Park, 2013; Li and Wu, 2015), batteries (Li et al., 2015; Bak et al., 2016; Kaur et al., 2018), supercapacitors (Le et al., 2011; Akhavan, 2015)], sensors (Shao et al., 2010; Wang and Arash, 2014; Chatterjee et al., 2015), catalysis (Han et al., 2017; Hu et al., 2017), electronics (like liquid crystal displays (Novoselov et al., 2005; Lin et al., 2015; Basu et al., 2016; Narayan et al., 2016), touch panels (Das and Prusty, 2013; Liu et al., 2014; Katkov and Osipov, 2017), electromagnetic interference shielding (Eswaraiah et al., 2011; Thomassin et al., 2013; Song et al., 2014; Cao et al., 2015), environmental remediation (Bi et al., 2012; Qin and Brosseau, 2012; Chabot et al., 2014), and so on. Clearly, there will be an increasing demand for high quality graphene.

Currently, reduction of graphene oxide or simultaneous exfoliation and reduction of graphite oxide are regarded to be most promising for large scale production of chemically derived graphene, which can be defined as reduced graphene oxide (rGO). In general, there are mainly two strategies used for graphene oxide reduction: chemical reduction and thermal reduction, both of which are facing certain challenges. In chemical reduction methods, the reduction efficiency is unsatisfactory, requiring long treating time and yielding rGO with oxygen content usually higher than 15% (Stankovich et al., 2007; Shao et al., 2010; Mattevi et al., 2011; Park et al., 2011b; Xiao et al., 2013; Zhang et al., 2013; Wen et al., 2014). In thermal reduction process, long-time high-temperature treatment is not only energy consuming but also results in fragmentation of graphene and lots of structural defects (Jones et al., 2002; Chen and Yan, 2010; Chen et al., 2010; Gao et al., 2010; Lin et al., 2010; Zhu et al., 2010b; Zhang et al., 2011).

Microwave heats materials directly through dielectric loss rather than heat convection as in the conventional heating method, and hence can enable fast heating and selective heating. It is regarded to be promising in shortening the reaction time and yielding hot spots with extraordinary high temperature (Thostenson and Chou, 1999; Kappe, 2004; Tompsett et al., 2006; Schwenke et al., 2015). As such, material scientists have devoted much effort to explore the potential application of microwave in overcoming the current problems and facilitating the fabrication of high-quality rGO in large-scale. To this end, we present an up-to-date critical survey of literature relevant to MW-assisted production of rGO. This brief review is organized as follows. First, techniques for characterizing quality of as-obtained rGO are discussed. Next, we provide a comprehensive summary of microwave-assisted chemical reduction of graphene oxide, microwave-assisted thermal reduction of graphene oxide, and microwave-assisted simultaneous thermal exfoliation and reduction of graphite oxide, respectively.

## Techniques for Characterizing Quality of rGO

Generally, X-ray diffraction, X-ray photoelectron spectroscopy, Raman spectroscopy, electrical conductivity, thermal gravity analysis are common techniques used for characterizing reducing efficiency and quality of rGO.

### X-Ray Diffraction

The X-ray diffraction (XRD) pattern of graphene/graphite oxide usually shows a characteristic diffraction peak at 2θ = 10.9° with the corresponding d-spacing of 0.81 nm (Stobinski et al., 2014; Strankowski et al., 2016), due to insertion of hydroxyl and epoxy groups between the carbon sheets. After reduction, two types of XRD spectra may be obtained: (1) The XRD pattern with no peaks, suggesting the complete reduction and the formation of single-layered rGO (Hassan et al., 2009). (2) The XRD pattern showing a broad peak around 26.5° (Zedan et al., 2010; Liu et al., 2011; Park et al., 2011b; Wang et al., 2011; Pokharel et al., 2014). Since the oxygen-containing functional groups could lead to a reduction in crystallinity, the higher the broad peak's intensity is, the higher the crystallinity degree is, and the better graphene/graphite oxide is reduced.

### X-Ray Photoelectron Spectroscopy

X-ray photoelectron spectroscopy (XPS) could help to obtain qualitative and quantitative analysis results of the content of oxygenated groups. For example, the carbon/oxygen atomic ratio (C/O ratio) could be evaluated from the areas of C1s and O1s peaks and the atomic sensitivity factor. Usually, the C/O ratio of graphene oxide is ~2.2–2.7 (Stankovich et al., 2007; Chen et al., 2010; Park et al., 2011a; Li et al., 2013; Wen et al., 2014; Han et al., 2015), attributed to the abundant oxygen-containing functional groups introduced during oxidation of graphite. Further, the C1s peak can be decomposed into three peaks: C = C (284.7 eV), C-O (286.9 eV), C = O (287.77 eV) (Zhao et al., 2014), through the areas of which existing statuses of oxygen could be analyzed in a quantitative manner.

### Raman Spectroscopy

The D band around 1,350 cm^−1^ in Raman spectra could reflect the disorder degree of the crystal structure of carbon while the G band around 1,580 cm^−1^ represents a first-order scattering E_2g_ vibration mode for characterizing the sp2 bond structure of carbon. I_D_/I_G_ is the ratio of D band intensity to G band intensity, which could be used to evaluate the quality of the graphene structure. The higher I_D_/I_G_ is, the more defects of C atom crystal there are. In the Raman spectrum of graphene, the D-band peak could not be observed. However, the I_D_/I_G_ of graphene oxide could be as high as ~0.8–1 (Chen et al., 2010; Lin et al., 2010; Liu et al., 2011; Park et al., 2011a; Zhao et al., 2014). Besides, the number of rGO layers can be estimated by observing the shape and position of the 2D peak in Raman spectra which is the second-order two-phonon process. For example, single-layer graphene exhibits a single, sharp 2D band located below 2,700 cm^−1^, while bilayer sheets have a broader 2D peak around 2,700 cm^−1^, and sheets more than five layers have a broad 2D peak above 2,700 cm^−1^ (Hassan et al., 2009).

### Electrical Conductivity

Though the conductivity of graphene is as high as 1 × 10^8^ S m^−1^, that of graphene oxide is about 0.02–0.07 S m^−1^ (Li et al., 2010; Zhu et al., 2010a; Park et al., 2011a) due to the existence of oxygen-containing functional groups which disrupted sp2 bonding networks. During the reduction process, electrical conductivity can be recovered by restoring the p-network as oxygen-containing groups leave, leading to a conductivity improvement by 3–6 orders of magnitude (Chen et al., 2010; Dreyer et al., 2010; Zhao et al., 2014; Han et al., 2015). Therefore, the restoration degree of conductivity can indirectly reflect the reduction degree and quality of rGO.

### Thermal Gravity Analysis

Graphene/Graphite has a very good thermal stability even when being heated up to 900°C (Bastiurea et al., 2015). For graphene oxide, it starts to have significant mass loss from ~200°C due to the decomposition of labile oxygen functional groups leads to poor thermal stability (Hassan et al., 2009; Chen et al., 2010; Zhu et al., 2010a; Gannavarapu et al., 2018). Therefore, the removal of oxygen-containing functional groups can help to restore its thermal stability. The significant mass loss temperature of rGO usually happens around 500–800°C, depending on the reduction degree of rGO.

## Microwave-Assisted Production of Reduced Graphene Oxide

Methods for producing rGO could generally be categorized into chemical reduction of graphene oxide, thermal reduction of graphene oxide, and simultaneous thermal exfoliation and reduction of graphite oxide. Here, we would also discuss microwave-assisted production of rGO in these three categories separately.

### Microwave-Assisted Chemical Reduction of Graphene Oxide

Strong reducing reagents have been widely used to reduce graphene oxide. However, the reduction process is very slow. For example, hydrazine hydrate enabled reduction of graphene oxide in the oil bath for 12 h and 24 h resulted in a C/O ratio about 5 (Park et al., 2011b) and 10.3 (Stankovich et al., 2007), respectively. Microwave irradiation was found to be capable of accelerating the reducing rate significantly. For example, Hassan et al. (2009) reported microwave assisted hydrazine hydrate reduction of graphene oxide. After microwave treatment for 2 × 30 s (on for 10 s, off and stirring for 20 s), the I_D_/I_G_ in Raman spectra was 0.1–0.12, indicating a high reduction degree even after such a short treating time This was confirmed by the improved thermal stability, showing no significant mass loss up to 750°C. Elazab et al. (2015) obtained Pd/Fe_3_O_4_ nanoparticles supported on graphene nanosheets by a one-pot microwave heating with the existence of hydrazine hydrate. After microwave irradiation for 2 min, the C/O of rGO was determined to be 8.1. Kumar et al. (2015) reported microwave-enhanced chemical reduction of graphene oxide using HI/CH_3_COOH as reducing reagent. They found the effect of 4 h microwave irradiation was comparable to 48 h conventional reaction process.

**Figure 1:**
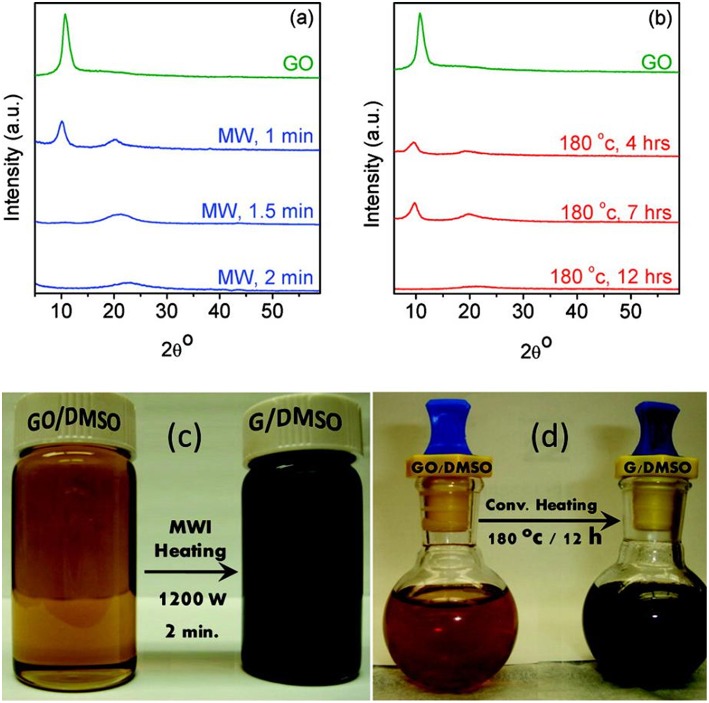
**(a)** XRD pattern of graphene oxide after microwave reduction for 1, 1.5, and 2 min. **(b)** thermal reduction after 4, 7, and 12 h. Digital camera images of graphene oxide and graphene colloidal suspensions in DMSO for **(c)** microwave and **(d)** thermal syntheses. [Reprinted with permission from Zedan et al. (2010), copyright 2010 American Chemical Society].

Since strong reducing regents are very much expensive and hazardous, organic solvents have also been explored to reduce graphene oxide. Zedan et al. (2010) reported MW-assisted reduction of graphene oxide in dimethyl sulfoxide (DMSO), a solvent with high microwave absorbing capability. The reduction degree of the sample prepared from 2 min MW irradiation at 1,200 W is even much higher than that of the sample derived from 7 h conventional heating treatment, as evidenced by XRD results of both samples shown in Figures 1a,b. After 2 min of MW irradiation in DMSO, the color of the product changed to black (Figure 1c), and the XRD pattern showed no characteristic peak (10.9°) of GO, indicating that most of the GO has been converted into rGO. In contrast, under conventional heating at 180 °C, an apparent peak located at 10.9° was found even after 7 h treatment. It was not until 12th h that the GO was completely reduced, as can be seen from the disappearance of the peak at 10.9° in the XRD pattern and the color change of the sample to black (Figure 1d). Chen et al. (2010) reported the microwave-enhanced chemical reduction of graphene oxide to rGO in a mixed solution of N, N-dimethylacetamide (DMAc) and water without any additional reductant under nitrogen purge. The XRD pattern of the graphene oxide showed a wide peak, indicating the damage of the regular crystalline pattern of graphite during the oxidation. After microwave treatment for 2 min × 5 times (2.45 GHz, 800 W), no peak was observed indicating microwave-assisted formation of single-layered graphene. XPS results showed that C/O ratio changed from 2.09 to 5.46, confirming that the removal of oxygen containing groups happened. However, the I_D_/I_G_ in Raman spectra changed from 0.95 to 0.96, suggesting that the reduction led to an increase of aromatic domains of smaller overall size in graphene. Similarly, Liu et al. (2011) also reported an increase of the product's I_D_/I_G_ in Raman spectra from 0.80 to 0.94 after being treated by microwave at 750 W for 2 min in N, N-dimethylacetamide (DMF) solution. Zhao et al. (2014) applied DMF to reduce graphite oxide under microwave irradiation at 100 W for 30 min. The XPS results showed that C/O ratio rose from 2.13 to 6.13 while Raman results showed that I_D_/I_G_ remained almost unchanged.

The characteristics of the as-obtained rGO products derived from different microwave-assisted chemical reduction methods are summarized and listed in Table 1. Since microwave heat rapidly, microwave-assisted rate enhancement in chemical reduction of graphene oxide has been widely observed, with most of the reaction time <10 min. However, as in the conventional heating method, the as-obtained rGO is still unsatisfactory, facing the same problem of containing a high oxygen content and a high I_D_/I_G_ value when organic solvent was used as the reducing reagent. Microwave-enhanced quality of chemically reduced rGO is yet to be explored. Also, since the type and amount of reducing agents were different in different work, it is difficult to draw a conclusion regarding the effect of microwave power and treating time here.

**Table 1:** Characteristics of rGO products derived from different microwave-assisted chemical reduction.

	**Experiment conditions**					**Results**					**References**
	**Frequency**	**MW power**	**MW time**	**Atmosphere**	**Reducing agent**	**XRD (Peak location)**	**XPS (C/O)**	**Raman (I_**D**_/I_**G**_)**	**Conductivity (S m^**−1**^)**	**TGA (significant mass loss T)**	
1	2.45 GHz	1,000 W	1 min	Air	hydrazine hydrate	No peak		0.12		750°C	Hassan et al., 2009
2	2.45 GHz	250 W	10 min	Air	hydrazine hydrate	~15°	8.1				Elazab et al., 2015
3	2.45 GHz	300 W	10 min	Air	hydrazine hydrate		5.28				Li et al., 2013
4	2.45 GHz	1,200 W	2 min	Air	DMSO	~24°					Zedan et al., 2010
5	2.45 GHz	800 W	10 min	N_2_	DMAc	No peak	5.46	0.96	200	~500°C	Chen et al., 2010
6	2.45 GHz	750 W	2 min	Air	DMF			0.94			Liu et al., 2011
7	2.45 GHz	100 W	30 min	Air	DMF	13.4°	6.13	0.95	29.9		Zhao et al., 2014

### Microwave-Assisted Thermal Reduction of Graphene Oxide

In conventional thermal reduction process, long-time high-temperature treatment in a protecting atmosphere (e.g., N_2_/Ar) is usually required, which is not only very energy consuming but also leads to the formation of defects in the graphene basal plane because of the evolution of the oxygen functional groups during reduction, like nanoscopic holes brought by carbon loss as CO or CO_2_ and Stone-Wales types of defects lead by rearrangement of carbon atoms in the graphene basal plane. Besides, the reducing efficiency is always suppressed by the highly stable ether and carbonyl groups formed between the oxygen functional groups.

**Figure 2:**
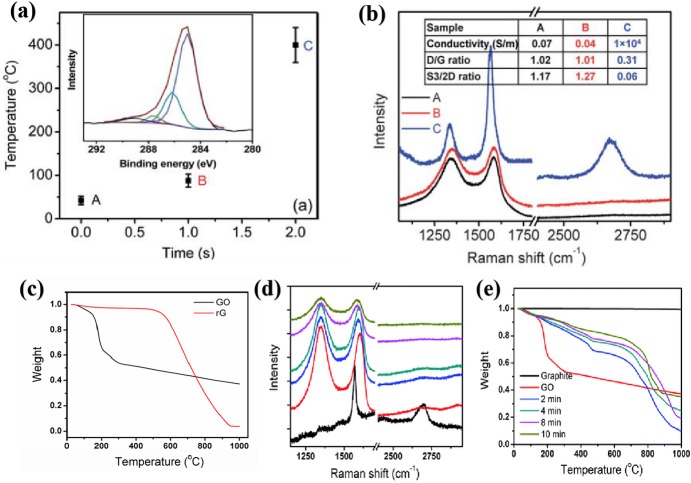
**(a)** The temperature rises of graphite oxide film under 6.425 GHz, 500 W MW within 2 s. Inset is XPS data of rGO. **(b)** Raman spectroscopy of graphite oxide at (A) before MW; (B) imminent to transition state; (C) after being reduced to graphene, respectively. **(c)** The TGA data of GO and rGO reduced by 6.425 ± 1.150 GHz MW. Raman **(d)** and TGA **(e)** characteristics of graphite, graphite oxide and different types of rGO samples by 2.45 GHz MW irradiation. [Reprinted with permission from Li et al. (2010), copyright 2010 The Royal Society of Chemistry].

In 2010, Li et al. (2010) for the first time reported a microwave-assisted thermal reduction of graphene oxide. Specifically, they made the graphene oxide into a free-standing film via filtration membrane, and then treated it with a microwave irradiation with a frequency of 6.425 ± 1.150 GHz at a power of 500 W without purging any inert gas. As shown in Figures 2a,b, after 1 s irradiation, the temperature rose only a little and no distinct difference were found in the electrical properties and the Raman spectra of the sample. However, after 2 s irradiation, the surface temperature of the graphene oxide film ramped up to 400°C and the conductivity of the graphene oxide film increased from 0.07 to 1 × 10^4^ S m^−1^; the I_D_/I_G_ in Raman spectra decreased from 1 to 0.3; the mass loss temperature increased from 181 to 668°C (Figure 2c). In addition, they found that in the MW treatment process, fierce degassing and a high gas pressure inside the graphene oxide was generated, which was beneficial for exfoliating the graphene oxide into thinner graphene sheets. Afterwards, they also applied a household MW oven (with a frequency of 2.45 GHz and a power of 1,000 W) to treat the free-standing graphene oxide film. They found that there was an optimal irradiation time of 8 min as evidenced by Raman spectra (Figure 2d) and TGA results (Figure 2e). Longer treatment time (e.g., 10 min) caused lower mass loss temperature (from 700°C to around 800°C), which could possibly be attributed to the oxidation by air when there was no protecting gas.

**Figure 3:**
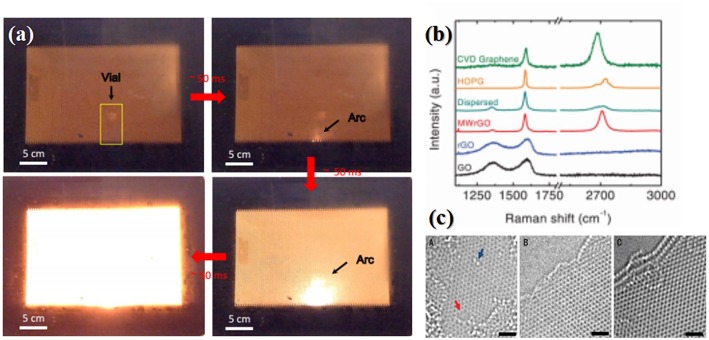
**(a)** Digital picture showing the formation of arcs during graphene oxide microwaving. **(b)** Raman spectra of microwave-reduced GO (MW-rGO) and other different rGO samples. **(c)** HR-TEM of MW-rGO nanosheets. (A) single-layer rGO presenting high density of defects. HR-TEM of (B) Bilayer and (C) trilayer MW-rGO showing highly ordered structure. The red arrow denotes a hole; the blue arrow indicates an oxygen functional group, scale bars, 1 nm. [Reprinted with permission from Voiry et al. (2016), Copyright 2016 American Association for the Advancement of Science].

Considering the weak microwave absorption of graphene at low frequency, while 2.45 GHz and 915 MHz are most commonly used in industry, Voiry et al. (2016) introduced a conventional mild annealing process before the microwave irradiation process. Firstly, the single-layered graphene oxide solution was slowly injected into an aqueous solution that contained 1 wt% CaCl_2_, which helps to coagulate GO nanosheets and promote solidification via bridging with the oxygen-containing groups from two GO nanosheets, yielding uniform and continuous gelatinous GO. The coagulated graphene oxide was then washed with DI water and dried, which was subsequently annealed at 300°C for 1 h under Argon to yield mildly reduced graphene oxide that was capable of absorbing microwaves at 2.45 GHz effectively. After 1–2 s long microwave irradiation in microwave oven at 1,000 W in argon, the efficient absorption of microwave led to rapid heating of the mildly reduced GO and consequently brought large arcing as shown in Figure 3a, causing desorption of oxygen functional groups and reordering of the graphene basal plane. As such, the as-obtained rGO exhibited pristine CVD graphene-like features in the Raman spectrum showing sharp G an 2D peaks and a nearly absence of D peak. In contrast, the rGO derived from conventional thermal reduction remains highly disordered, as indicated by the presence of an intense and broad disorder D band and the absence of the 2D band in the Raman spectra (Figure 3b). The XPS results were well in agreement with the Raman results. Owning to the large arcing which may possibly resulted in an extremely high temperature, the oxygen content of the MW-rGO was about 4%, lower than that theoretically predicted for rGO after annealing at 1,500 K (15–25%). Aberration corrected HR-TEM (Figure 3c) also suggested a highly ordered structure, which suggests that there is some reorganization of the carbon bonding during microwave reduction, along with removal of oxygen facilitated by achieving exceptionally high temperatures.

**Figure 4:**
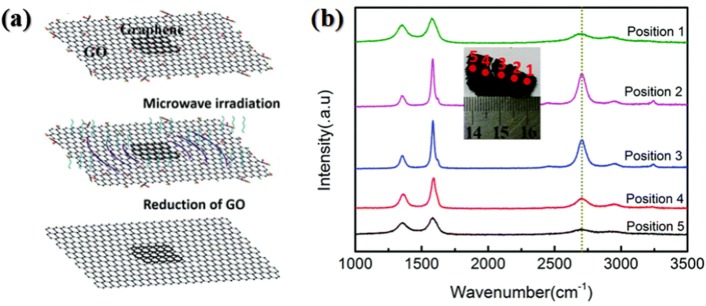
**(a)** Schematic diagram of the fabrication process of graphene (rGO paper)-triggered microwave reduction. **(b)** Raman spectra of five measurement points on a strip MW-rGO sample. [Reprinted with permission from Jiang et al. (2018), copyright 2018 The Royal Society of Chemistry].

Jiang et al. (2018) further modified Voiry's strategy and developed a triggered microwave-assisted reduction of graphene oxide. In their work, 1 wt% CaCl_2_ aqueous solution was used to treat one side of the filter paper while graphene oxide was dropped onto the other side of the filter paper. Subsequently, graphene oxide was treated with CaCl_2_ solution and then washed with DI water, dried in air and pulled out from the filter paper. Finally, a small piece of rGO “paper” obtained by thermal annealing was placed on the as-obtained large graphene oxide paper to act as a trigger, as shown in Figure 4a. After 800 W microwave irradiation for 2 s in air, arc discharge process started. The irradiation was maintained for extra 3–5 s and high-quality microwave reduced graphene oxide paper was obtained. In XPS spectrum, the characteristic peak for C-O vanished, suggesting the significant removal efficiency of oxygen-containing group. Raman spectra of MW-rGO showed sharp G and 2D bands, and low-intensity D band. In contrast, the Raman spectra of rGO derived from conventional thermal reduction at 800°C showed a strong D band and none 2D band, indicating microwave heating is very beneficial for recovering the highly ordered graphene-like structures which was also confirmed by the conductivity. The sheet resistance of the MW-rGO was measured to be about 40 Ω cm^−2^, far less than that (about 796 Ω cm^−2^) of rGO prepared by 800°C annealing. They further examined the Raman spectra of graphene-triggered microwave reduction at different positions, and found the part of graphene oxide located nearest to the trigger was not reduced the best, as shown in Figure 4b. Hence, they proposed that the microwave reduction can be not only governed by a simple thermal reduction process but the result of the thermal effect and arc discharge (Jiang et al., 2018).

**Figure 5:**
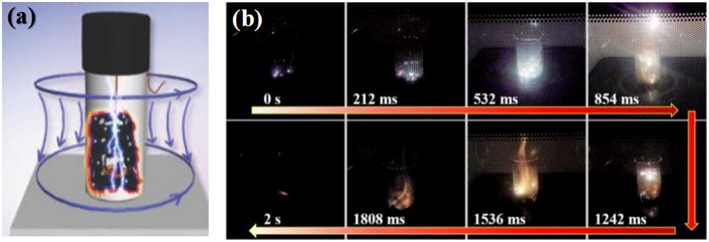
**(a)** Schematic diagram of the microwave combustion system. **(b)** Digital picture showing the formation of arcs during microwave process. [Reprinted with permission from Wan et al. (2018), copyright 2018 John Wiley and Sons].

Instead of putting a small piece of graphene as the trigger, Wan et al. (2018) put a fully bended copper wire inside graphene oxide to absorb 2.45 GHz microwave irradiation intensively and hence trigger immediate arcs and fire to reduce the graphene oxide, as shown in Figures 5a,b. Microwave treatment is applied for 2 s one time. The decreasing D band in Raman spectra under different microwave treating times indicate that high crystalline graphene was obtained after microwave treatment.

Low-temperature reduction of graphene oxide is of great importance when graphene oxide film was deposited on glass or plastic substrates. Han et al. (2015) demonstrated microwave-assisted low-temperature thermal reduction of few-layered graphene oxide film. Specifically, they put the graphene oxide film at the center of the highest magnetic field in a single mode microwave reactor which was operated at 2.45 GHz with a power of 42 W to ensure that the temperature was below 250°C during the whole process. After 5 min treatment in the air, I_D_/I_G_ in the Raman spectrum of the product decreased from 2.89 to 1.56 and C/O derived from XPS results increased from 7.8 to 17.5. In addition, microwave-enabled exfoliation leading to thinner pieces was also observed in their work.

The characteristics of the as-obtained rGO products derived from different microwave-assisted thermal reduction of graphene oxide methods are summarized and listed in Table 2. Arc discharge caused by microwave irradiation is very beneficial for obtaining high-quality rGO, as evidence by the short reaction time, the low I_D/_I_G_ values, the high C/O values and the conductivity values of most as-derived rGO. It is worth to note that rGO containing only 4% oxygen and exhibiting pristine CVD graphene-like features in the Raman spectrum was successfully fabricated via this reducing strategy. Besides, owning to the ultra-rapid treating process, protecting atmosphere is not always necessary. However, too long treatment time would result in re-oxidation and consequently rGO with worse quality when the reduction is done in the air. Since the amounts of GO samples and the microwave heating chambers in different work were different, which would affect the power intensity imposed on the GO, it is also difficult to draw a conclusion regarding the effect of microwave power here.

**Table 2:** Characteristics of rGO products obtained by different microwave-assisted thermal reduction.

	**Experiment conditions**				**Results**					**References**
	**Frequency**	**MW power**	**MW time**	**Atmosphere**	**XRD (peak location)**	**XPS (C/O)**	**Raman (I_**D**_/I_**G**_)**	**Conductivity**	**TGA (significant mass loss T)**	
1	2.45 GHz	42 W	5 min	Air		17.5	1.56	6 × 10^3^ Ω cm^−2^		Han et al., 2015
2	6.425 GHz	500 W	2 s	Air			0.3	1 × 10^4^ S m^−1^	668°C	Li et al., 2010
3	2.45 GHz	800 W	5–7 s	Air	25.6°		0.32	40 Ω cm^−2^		Jiang et al., 2018
				N_2_			0.14			
4	2.45 GHz	1,000 W	1–2 s	Ar			< 0.1			Voiry et al., 2016
5	2.45 GHz	1,000 W	8 min	Air					800°C	Li et al., 2010
6	2.45 GHz		2–40 s	Air	No peak					Wan et al., 2018

### Microwave-Assisted Simultaneous Exfoliation and Reduction of Graphite Oxide

**Figure 6:**
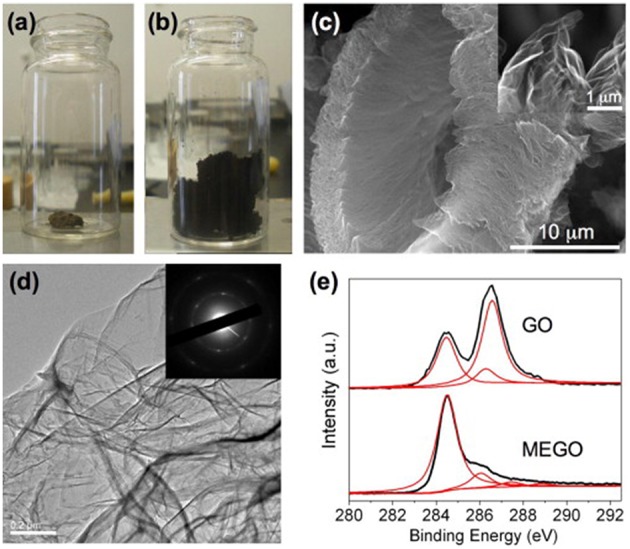
Optical photos of graphite oxide before **(a)** and after **(b)** treatment in a microwave oven for 1 min. **(c)** Typical SEM image of as prepared microwave exfoliated graphite oxide (MEGO) by microwave irradiation with a high magnification SEM image in the inset showing the crumpled MEGO sheets. **(d)** Typical TEM image of the MEGO and the corresponding electron diffraction pattern. **(e)** XPS C1s spectra of GO and MEGO. [Reprinted with permission from Zhu et al. (2010a), Copyright 2010 Elsevier].

Beside of direct reduction of graphene oxide to yield rGO, thermal shock like flash-assisted simultaneous exfoliation and reduction of graphite oxide at temperatures up to 1,050°C has also been used. Theoretically, microwave heating which is well-known for its rapid heating feature is very promising in enabling simultaneous exfoliation and reduction of graphite oxide. In 2010, Zhu et al. (2010a) treated graphite oxide powder in microwave oven at 700 W for 1 min in ambient atmosphere. Large volume expansion as shown in Figures 6a,b accompanied with violent fuming followed by sparking was observed. They found that a minimum power of 280 W was required to expand the graphite oxide powder successfully, which was probably due to the insulating feature that resulted in inferior microwave absorption ability. After microwave treatment, the as-obtained rGO had a worm-like morphology, in which many regions are wrinkled and folded (Figures 6c,d). XPS results showed that C/O increased from 0.79 to 2.75 (Figure 6e). XRD spectrum showed a broad peak centered at 2θ = 25°. The conductivity was measured to be about 274 S m^−1^.

Under microwave irradiation, graphite oxide itself might not be heated to a high enough temperature to cause its exfoliation and reduction efficiently due to its inferior microwave absorption ability. Scientists have tried several ways to come over this problem.

**Figure 7:**
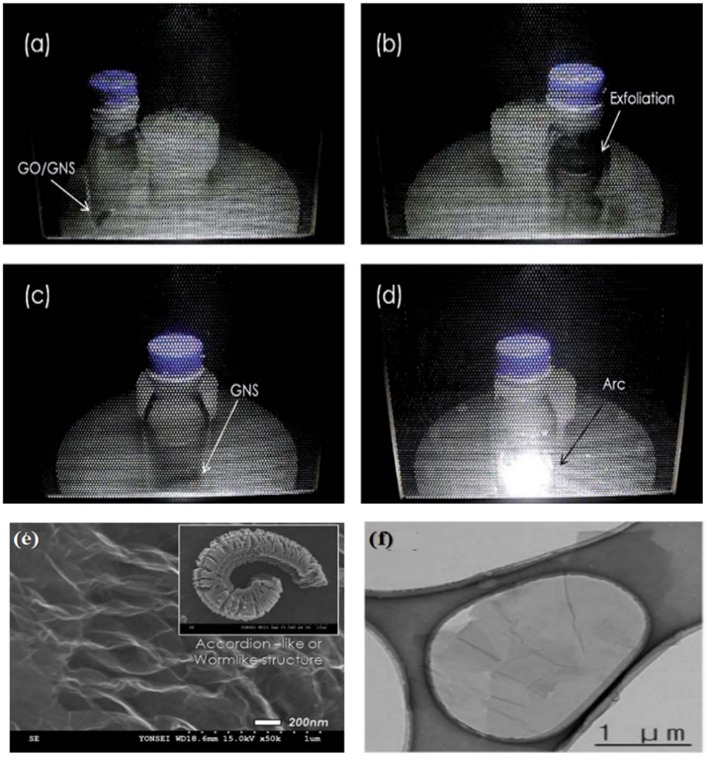
Digital images of the sequence of the exfoliation process during solid-state microwave irradiation synthesis: **(a)** graphite oxide/graphene nanosheets (GNS) mixture powder, **(b)** exfoliation of graphite oxide/GNS powder. **(c)** GNS after exfoliation and **(d)** arc evolution from GNS. **(e)** SEM images of GNS by solid-state microwave irradiation synthesis. **(f)** HR-TEM images of GNS. [Reprinted with permission from Park et al. (2011a), copyright 2011 The Royal Society of Chemistry].

Park et al. (2011a) mixed graphite oxide powder with 10% graphene nanosheets to realized efficient exfoliation and reduction under microwave irradiation. The temperature rising rate was estimated to be 2,000°C/min, which was high enough to build up pressure to overcome the van der Waals forces between the graphene sheets in graphite oxide for exfoliation. Huge volume expansion was observed after 10 s, followed by arc after 40 s. SEM images (Figure 7) showed that the prepared rGO also had a worm-like structure composed of ultra-thin sheets, which is different from randomly agglomerated and wrinkled sheets prepared from solution-based reduction syntheses. HR-TEM images suggested the presence of single or a few layers of graphene nanosheets as shown in Figure 7. They further compared the effect of reduction atmosphere on the product quality. The rGO prepared in air, Ar, H_2_+Ar (1:9) showed a C/O ratio of 3.6, 11.45, 18.5, respectively and I_D_/I_G_ values of 0.853, 0.842, and 0.785, respectively in Raman spectra, respectively. The rGO prepared in Ar, H_2_+Ar showed a conductivity of 7.41 × 10^2^ and 1.25 × 10^3^ S m^−1^, respectively. Hydrogen containing atmosphere is the most effective because H_2_O was formed instead of CO_2_, which prevents the formation of vacancies and defects due to the loss of carbon atoms.

Wen et al. (2014) applied a sequential chemical reduction and microwave exfoliation and reduction of graphite oxide to produce rGO. Specifically, graphite oxide powder was treated by NaBH_4_ at 95°C for 2 h, and subsequently made into a film, which was microwave irradiated at 5.8 GHz with a power of 1,400 W for 10 min in N_2_. After NaBH_4_ treatment, a C/O value of 3.84, an I_D_/I_G_ value of 1.05 in Raman, and a conductivity of 1.41 × 10^2^ S m^−1^ were obtained. During the microwave treatment, the sample expanded to a loose floc and transmitted bright orange light, leading to ultrathin sheets with a C/O ratio of 10.33, an I_D_/I_G_ ratio of 1.07 in the Raman spectrum and a conductivity of 1.19 × 10^4^ S m^−1^. Sole microwave irradiation at 5.8 GHz with a power of 1,400 W for 10 min without a preceding chemical reduction process gave yield to a C/O of 7.24 and an I_D_/I_G_ of 0.89 in Raman spectrum. This demonstrated that solid-state microwave irradiation not only removed oxygen-containing groups, but also repaired defects on the graphene sheets while NaBH_4_ though effective for removing oxygen-containing groups may induce more defects. Further, through XPS analysis of content of C-O and C = O groups, they found that microwave irradiation removed C = O more readily than C-O while the chemical reduction method removed C-O more efficiently.

Pokharel et al. (2014) developed a multi-step reduction technique to obtain rGO from graphite oxide. In the first step, graphite oxide with a C/O of 0.83 was exfoliated within 1 min under microwave irradiation at 2.45 GHz with a power of 1,000 W in N_2_ without becoming red. The expanded graphite oxide had a C/O of 2.42. However, it was so loose that microwave could not concentrate on a definite area for further reduction of graphite oxide. Hence, in the second step, the expanded graphite oxide was collected and then exposed to additional microwave irradiation for 1 and 2 min, resulting in rGO showing a C/O ratio of 5.78 and 10.39, respectively.

The characteristics of the as-obtained rGO products derived from different microwave-assisted simultaneous thermal exfoliation and reduction of graphite oxide methods are summarized and listed in Table 3. As can be seen from the C/O values, I_D_/I_G_ values, mass loss temperatures and conductivities, simultaneous exfoliation and reduction was not able to yield rGO with as high quality as those obtained from thermal reduction of graphene oxides. However, when combined with reducing atmosphere (i.e., H_2_) or pretreatment with strong reducing reagents (NaBH_4_), the reduction degree could be greatly enhanced.

**Table 3:** Characteristics of rGO products derived from different microwave-assisted simultaneous thermal exfoliation and reduction.

	**Experiment conditions**				**Results**					**Additional information**	**References**
	**Frequency**	**MW power**	**MW time**	**Atmosphere**	**XRD (peak location)**	**XPS (C/O)**	**Raman (I_**D**_/I_**G**_)**	**Conductivity (S m^**−1**^)**	**TGA (significant mass loss T)**		
1	2.45 GHz	700 W	1 min	Air	25°	2.75		274	~500°C		Zhu et al., 2010a
2	2.45 GHz	1,600 W	50 s	Air		3.6	0.853				Park et al., 2011b
				Ar		11.45	0.842	7.41 × 10^2^			
				Ar+H_2_		18.5	0.785	1.25 × 10^3^			
3	5.8 GHz	1,400 W	10 min	N_2_		7.24	~0.7	7.63 × 10^2^			Wen et al., 2014
						10.33	~1.07	1.19 × 10^4^		Pretreated by NaBH_4_, and Raman beacomes worse. Maybe the MW not be sufficient to repair the large amount of defects caused by NaBH_4_	
4	2.45 GHz	1,000 W	1 min	N_2_	~12&24°	2.42			~450°C		Pokharel et al., 2014
			2 min		~22°	5.78					
			3 min		26.3°	10.39					

## Perspectives

It is expected that the explored methods for microwave-assisted reduction of graphene/graphite oxide into high-quality rGO could be extended to fabrication of graphene composites. The microwave-induced arc facilitated short reaction time at high temperature is not only beneficial for getting high-quality rGO, but also promising in facilitating the formation of high-quality nanomaterials (such as metal nanocrystals, metal oxides nanocrystals, and so on) with advanced performance. In addition, the methods could be extended to fabrication of other conductive materials that could trigger sparks under microwave irradiation, like graphene does.

## Author Contributions

All authors listed have made a substantial, direct and intellectual contribution to the work, and approved it for publication.

### Conflict of Interest Statement

The authors declare that the research was conducted in the absence of any commercial or financial relationships that could be construed as a potential conflict of interest.
